# Benchmarking methods for detecting differential states between conditions from multi-subject single-cell RNA-seq data

**DOI:** 10.1093/bib/bbac286

**Published:** 2022-07-25

**Authors:** Sini Junttila, Johannes Smolander, Laura L Elo

**Affiliations:** Turku Bioscience Centre, University of Turku and Åbo Akademi University, Tykistökatu 6, 20520 Turku, Finland; Turku Bioscience Centre, University of Turku and Åbo Akademi University, Tykistökatu 6, 20520 Turku, Finland; Turku Bioscience Centre, University of Turku and Åbo Akademi University, Tykistökatu 6, 20520 Turku, Finland; Institute of Biomedicine, University of Turku, 20520 Turku, Finland

**Keywords:** single cell, RNA sequencing (RNA-seq), differential expression

## Abstract

Single-cell RNA-sequencing (scRNA-seq) enables researchers to quantify transcriptomes of thousands of cells simultaneously and study transcriptomic changes between cells. scRNA-seq datasets increasingly include multisubject, multicondition experiments to investigate cell-type-specific differential states (DS) between conditions. This can be performed by first identifying the cell types in all the subjects and then by performing a DS analysis between the conditions within each cell type. Naïve single-cell DS analysis methods that treat cells statistically independent are subject to false positives in the presence of variation between biological replicates, an issue known as the pseudoreplicate bias. While several methods have already been introduced to carry out the statistical testing in multisubject scRNA-seq analysis, comparisons that include all these methods are currently lacking. Here, we performed a comprehensive comparison of 18 methods for the identification of DS changes between conditions from multisubject scRNA-seq data. Our results suggest that the pseudobulk methods performed generally best. Both pseudobulks and mixed models that model the subjects as a random effect were superior compared with the naïve single-cell methods that do not model the subjects in any way. While the naïve models achieved higher sensitivity than the pseudobulk methods and the mixed models, they were subject to a high number of false positives. In addition, accounting for subjects through latent variable modeling did not improve the performance of the naïve methods.

## Introduction

Single-cell RNA-sequencing (scRNA-seq) can be used to quantify transcriptomes of thousands of single cells simultaneously. scRNA-seq experiments comprise multisubject, multicondition setups, in which each condition includes samples collected from multiple subjects, cell lines or other biological replicates, and the researchers want to investigate transcriptomic changes between the conditions. Obtaining a large enough number of samples is crucial to ensure that the discovered gene markers are prevalent in the subject groups or treatment conditions, and not only in single subjects or biological replicates.

The analysis workflow of multisubject, multicondition scRNA-seq data involves steps that are the same as in any scRNA-seq analysis. Quality control is important to remove poor-quality cells, such as doublets, empty droplets and dead cells [1]. Normalization aims to make the gene expression profiles of different cells more comparable by decreasing the technical bias caused by the library size and other confounding factors [2]. In cell type identification, each cell is given identity from the cell types that are present in the tissue. Data integration methods can be used to automate the identification of the same cell types across the samples [3, 4].

Once the cell types have been confidently identified from all the samples, the next step is to perform a differential state (DS) analysis between two or more conditions within each cell type separately. DS changes can be divided into several subtypes [5], including changes in the mean expression, which is commonly known as differential expression (DE). The other DS types model more subtle transcriptomic differences, such as the proportion of highly and lowly expressed cell populations. While virtually all methods have been designed to detect only changes in the average expression, single-cell method developers have recently started to pay attention to the other DS types as well [6, 7].

The classical statistical tests for DS testing in scRNA-seq data, such as the Wilcoxon rank-sum test, naïvely assume the samples are statistically independent. However, this is usually not the case in multisubject scRNA-seq data, where cells from the same subject often have more similar gene expression profiles, which causes an error in the statistical testing known as the pseudoreplicate bias [8]. To alleviate the pseudoreplicate bias, two approaches currently exist. The first approach is to use mixed models that model subjects as a random effect. The second approach is pseudo-bulk aggregation, which transforms scRNA-seq data into bulk-like data by aggregating gene counts within each cell type and subject. Both approaches have previously been shown to reduce the number of false positives [6, 8–10].

DE analysis in scRNA-seq data was first investigated in papers that did not address the issue of multisubject setup [11, 12]. Since then, a few papers have investigated the issue of multisubject, multicondition scRNA-seq DE analysis. However, there still remains a lack of consensus regarding the best approaches. The muscat simulator [6] was introduced to enable the simulation of multisubject, multicondition data based on reference data, and it also allows simulating other DS types with more subtle differences in addition to DE. The muscat R package also provides functions for several pseudobulk methods and mixed models. A more recent paper by Zimmerman *et al*. [8] compared several off-the-shelf mixed models, pseudobulk methods and naïve methods that do not model the subjects in any way using a limited simulation setup. The simulation was based on plate-based data with dropouts and not droplet data, such as chromium [13], which is currently the most popular scRNA-seq protocol and is generally not considered zero-inflated [14]. The authors recommended a mixed model based on the MAST statistical test [15] (MAST_RE) that accounts for the subjects as a random effect and claimed it was superior compared to the pseudobulk methods. Another recent paper by Squair et al. [10] compared naïve methods, pseudobulk methods and one mixed-model method (muscat_MM). Their comparison was not based on a simulation but on a comparison between paired scRNA-seq and bulk RNA-seq data. The ground truth for the bulk data was defined using two of the bulk DE tests, which could cause significant bias in the results. The comparison did not consider the recently introduced MAST model (MAST_RE) [8] or NEBULA, which is another recently introduced mixed model specifically designed for the DS analysis of multisubject scRNA-seq data [16].

To address the need for a better understanding of the relative performance of various naïve, pseudobulk, and mixed-model methods, we compared 18 different methods for DS analysis of multisubject scRNA-seq data. Our comparison included three mixed models (MAST_RE [8], muscat_MM [6] and NEBULA-LN [16]) that model subjects as a random effect, six pseudobulk methods (edgeR [17] and DESeq2 [18] with sum aggregation, Limma [19] and ROTS [20] with sum and mean aggregation), and five naïve methods (the popular Wilcoxon rank-sum test and four other methods from the Seurat R package [3]). The three mixed models have not been compared before in the same study. Additionally, we tested four latent variable methods from the Seurat R package that can be used to account for variables, such as batch effects in DS analysis. The suitability of the latent variable methods to account for different latent variables such as subjects in DS analysis is not yet fully understood. To compare the DS analysis methods, we first carried out a comprehensive simulation analysis based on two different simulation models. The performance was assessed using several gold-standard performance metrics: area under the receiver operating characteristic curve (AUROC), sensitivity, specificity, precision, F1-score and Matthew’s correlation coefficient (MCC). We estimated the proportion of false positives by performing a mock comparison between random groups using real data. Finally, we investigated the reproducibility of the methods by investigating the correlation between the obtained gene lists from different subsets of the same data.

## Materials and methods

### Methods for detecting DS

In total, we considered 18 DS analysis methods in our comparison (Table 1). These methods belong to two broad categories: pseudobulk methods and single-cell methods. The pseudobulk methods aggregate count values from each sample and cell type (cluster) to create data that can be analyzed using the same methods as bulk RNA-seq data, maintaining the same number of genes but reducing the number of cells to the number of samples in the gene expression matrix. Single-cell methods assume that the data have been normalized at the single-cell level, and the DS analysis is carried out using the normalized data directly. The single-cell methods can be further divided into two subcategories: mixed models and naïve methods. The mixed models model the subjects as a random effect, whereas the naïve models assume that all the cells are statistically independent and do not model the subjects in any way. In addition, we considered a third type of single-cell method from the Seurat R package, the latent variable model, that tests whether the difference in gene expression between the groups can be explained by the difference in one or multiple latent variables. These methods were designed to account for batch effects or other confounders in the data.

**Table 1:** Details of the methods for the differential state analysis of scRNA-seq data compared in this study

	**Pseudobulk methods**				**Single-cell methods**							
	Pseudobulk methods that require built-in normalization		Pseudobulk methods that can be used with any normalization		Mixed models accounting for subjects as a random effect			Naïve methods that do not model subjects	Methods that have the option to use latent variables to correct for batches, etc.			
Method name	DESeq2	edgeR	Limma	ROTS	MAST_RE	muscat_MM	NEBULA-LN	wilcoxon	MAST	LR	negbinom	poisson
Normalization	Median of ratios	TMM	TMM + voom	TMM + CPM + log2	No default (Log normalize)	Log normalize	Normalization factors from library sizes	Log normalize	Log normalize	Log normalize	Log normalize	Log normalize
Statistical tests	Negative binomial generalized linear model	Negative binomial model + empirical Bayes procedure	Linear model + empirical Bayes procedure	Reproducibility optimized test statistic	Two-part hurdle model with random effect for subject	lme4 linear mixed model with voom weights	Negative binomial mixed model	Wilcoxon rank sum test	Two-part hurdle model	Logistic regression	Negative binomial generalized linear model	Poisson generalized linear model
R packages (normalization, test)	DESeq2	edgeR	edgeR, Limma	edgeR, ROTS	MAST	muscat	nebula	Seurat	Seurat, MAST	Seurat	Seurat	Seurat
Filtering	Nonexpressed genes	Nonexpressed genes	Nonexpressed genes	Nonexpressed genes	Number cells expressing gene < subjects	Number cells expressing gene <20, Number cells in sample < 10	Genes with counts per cell <0.005	Nonexpressed genes	Nonexpressed genes	Nonexpressed genes	Number cells expressing genes <3	Number cells expressing genes <3
Version	1.32.0	3.34.0	3.48.0	1.20.0	1.18.0	01-06-2000	01-01-2007	4.0.2	4.0.2	4.0.2	4.0.2	4.0.2
References	[18]	[17]	[17, 19]	[17, 20]	[15]	[6, 21, 22]	[16]	[23]	[15, 23]	[23]	[23]	[23]

The aggregation of the count values for pseudobulk methods can be performed using two approaches: cumulative summing of raw count values (sum) or averaging single-cell-normalized count values (mean). The sum aggregation is followed by bulk normalization, and it has achieved better performances in earlier studies than the mean aggregation [6]. A recent study by Thurman *et al.* [9] recommended the sum aggregation with DEseq2 for multisubject DS analysis, which is a popular statistical test for bulk RNA-seq DE analysis [18]. We selected DEseq2 and three other statistical tests, Limma, edgeR and ROTS [17, 19, 20] as a representation of the pseudobulk methods. In addition to performing the pseudobulk aggregation for all four statistical tests by the sum aggregation, we also tested the mean aggregation for two of the statistical tests (ROTS and Limma) that can be used with any normalization method. The sum and mean aggregated pseudobulk methods are denoted with _sum and _mean suffixes in the results, respectively.

Mixed models that account for the subjects as a random effect are gathering increasing interest. We included three mixed models in our comparison: MAST_RE, muscat_MM and NEBULA-LN. A recent paper by Zimmerman *et al.* [8] recommended for multisubject DS analysis a MAST model (MAST_RE) [15] that models the subjects as a random effect. The muscat R package includes a mixed model (muscat_MM), which uses the lme4 linear mixed model with voom weights [6, 21, 22]. NEBULA-LN is a recently introduced negative binomial mixed model designed for fast, multisubject DS analysis and estimation of co-expression between genes [16].

Seurat is a popular R package for scRNA-seq data analysis, including a wide array of statistical tests for DS analysis [3, 23]. These include naïve methods that do not model the subject in any way, such as the Wilcoxon rank-sum test, as well as models that can be used with ‘latent variables’ to account for different confounding factors during the statistical testing. The way in which the latent variable modeling is performed varies depending on the statistical test. The batch effect is the only confounder that is mentioned in the documentation, but the user can include an arbitrary number of latent variables in the *FindVariables* function. The four statistical tests of Seurat that support the use of latent variables are MAST, logistic regression, negative binomial generalized linear model (negbinom) and Poisson generalized linear model (Poisson). We included these four tests and their naïve versions in our comparison. In addition, we included the Wilcoxon rank sum test, which is the default method for DS analysis in Seurat. Other approaches for performing multisubject DS analysis, such as mixed models with random effects or pseudobulk methods, are not currently available in Seurat (version 4.1).

### Simulation of scRNA-seq data

Since it is in practice very difficult to ascertain which genes are differentially expressed between conditions in real scRNA-seq data, simulation is necessary to obtain an accurate benchmark. To simulate scRNA-seq count data, we used two different approaches. The first approach is based on a reference-free negative binomial generative model presented in the original study of one of the benchmarked tools (NEBULA). This approach can simulate DE and non-DE genes by controlling the average fold change between the groups but no other DS types. The second approach uses muscat, which is a recently introduced R package based on a reference-based negative binomial generative model that enables simulating multisubject, multicondition scRNA-seq data using real data as reference [6]. It can simulate genes of four different DS types and two non-DS types: changes in the mean expression (DE), changes in the proportions of low and high expression components (DP), changes in the differential modality (DM), changes in both the proportions and modality (DB), equivalent expression (EE), and equivalent expression at low and high components by an equal proportion (EP).

#### Simulation using a reference-free negative binomial generative model

We performed a reference-free negative binomial generative model simulation using the approach from the original paper of one of the benchmarked tools (NEBULA-LN) [16]. This simulation allowed tuning of the model parameters, including two overdispersion parameters (cell and sample) that create random variation in the gene expression levels between cells and samples, and the average number of cells per sample.

To generate gene expression data that included both non-DE and DE genes, we made changes to the original NEBULA simulation. As in the original simulation, we simulated non-DE genes by setting logFC = 0. Additionally, we simulated DE genes with logFC between 0.5 and 2.0. In total, our simulation included 1280 datasets, each containing 100 DE genes and 1900 non-DE genes. We simulated the 1280 datasets by adjusting five different parameters: the number of samples (6, 8, 10, 12, 14, 16, 18, 20, 30, 40), the average number of cells per sample (100, 500, 1000, 2000), the distribution for sampling the average number of cells (Poisson, negative binomial), cell overdispersion (0.05, 0.10, 0.20, 0.50) and sample overdispersion (0.1, 1, 10, 100). The average expression term in the generative model ranged from −4 to 2.

#### Simulation using a reference-based negative binomial generative model

In our simulation with muscat, we considered reference data from four studies (Kang [24], Kallionpää [25], Thurman [9] and Liu [26]), which are summarized in Table 2. The Kang dataset comprises peripheral blood mononuclear cells (PBMC) from lupus patients before and after treatment with interferon-β. The Kallionpää dataset includes PBMC cells from children that developed type I diabetes at a young age along with paired control samples. The Thurman dataset includes cells segregated from large and small airway surface epithelium of newborn cystic fibrosis (CF) and non-CF pigs. The Liu dataset includes PBMC cells from COVID-19 patients, patients with tropical infectious diseases and healthy subjects.

**Table 2:** Details of the reference datasets used in the simulation using a reference-based negative binomial generative model

	**Kang**	**Kallionpää**	**Liu**	**Thurman**
Number of replicates in simulation	8, 16	12	12, 16, 20	8
Tissue types	PBMC	PBMC	PBMC	Airway surface epithelium
Conditions	IFN-β-treated versus nontreated	T1D cases versus matched controls	COVID-19 versus healthy controls	CF versus non-CF
Organisms	Human	Human	Human	Pig
Number of control samples in original dataset	8	4	14	4
References	[24]	[25]	[26]	[9]

For each simulated dataset, we simulated three clusters with varying magnitudes of differences. 10% of the genes in each cluster were assigned a differential distribution (2.5% for each of the four differential distributions DE, DP, DM and DB). The relative log-fold-change (logFC) values were set to 0.5, 1 and 1.25 for clusters 1, 2 and 3, respectively. Using Kang data as reference, four datasets were simulated: 20 000 cells and 4 replicates per condition, 20 000 cells and 8 replicates per condition, 5000 cells and 4 replicates per condition and 5000 cells and 8 replicates per condition. One dataset was simulated using Kallionpää data as reference: 7500 cells and 4 replicates per condition. Using Liu data as reference, three datasets were simulated: 12 000 cells and 6 replicates per condition, 16 000 cells and 8 replicates per condition and 20 000 cells and 10 replicates per condition. One dataset was simulated using Thurman data as reference: 20 000 cells and 4 replicates per condition. Additionally, to investigate the impact of the number of cells and the number of samples on the performance, we extended the muscat simulation for the Liu dataset so that it included more variation in the number of cells per sample (500, 1000, 2000, 4000) and the number of subjects (8, 12, 16, 20, 24, 28, 32, 36, 40).

For Kang, Liu and Thurman reference data, we used the cell type annotation that was provided by the authors of the original studies in the muscat simulation. For Kallionpää data, we performed Seurat integration (v 4.0.3) with the default parameter values and used the resulting clustering in the muscat simulation.

#### Simulation of imbalanced distribution of cells across the samples

In both simulations, the datasets contained an almost even distribution for the number of cells between subjects. However, this assumption is not valid in many real situations, and a recent paper by Zimmerman *et al.* [8] suggested that especially the performance of pseudobulk methods deteriorates when a dataset has an uneven distribution for the number of cells. Therefore, we also simulated clusters that had a large variation in the number of cells between the samples. In the reference-free simulation, the random sampling of the number of cells was performed using two statistical distributions: Poisson for balanced distribution and negative binomial for imbalanced distribution. In the reference-based simulation, we randomly subsampled cells for all simulated datasets without replacement so that the proportion of the remaining cells in the samples varied with even intervals from 0.20 to 1. The subsampling was performed for each of the clusters separately and the proportions of remaining cells were chosen randomly for the samples.

### Performance evaluation

We performed receiver operating characteristic (ROC) curve analysis on the simulation results using the pROC R package [27]. As the predictor, we used the *P*-values, and as the response, the ground truth provided by the simulation on which genes had DS. Since the methods had different gene filtering strategies (Table 1), we only considered genes that were included by all methods.

While the AUROC is useful for assessing the performance so that the evaluation is not constrained to a specific *P*-value threshold, and it can be interpreted as measuring the accuracy of ranking positive genes higher than negatives, it is possible to achieve a perfect AUROC score with statistically insignificant *P*-values. To assess the ability of the methods to provide well-calibrated *P*-values, we also calculated the sensitivity, specificity, precision, F1-score and MCC of the methods using the false discovery rate (FDR) of 0.05 as a cutoff. Before adjusting the *P*-values for multiple comparisons, we excluded the genes that were not included by all the methods (Table 1).

### Average overlap to measure differential distribution of points between conditions

To measure the differential distribution of data points between two groups, we define a metric called the average overlap. We count in each group how many pseudobulk normalized data points (replicates) are within the range of the values of the other group, divide the counts by the number of samples in each group and then average the two ratios. Given two pseudobulk normalized gene expression vectors, ${\boldsymbol{x}}_{\boldsymbol{A}}=[{x}_{A1},\dots, {x}_{Am}]$ and ${x}_B=[{\boldsymbol{x}}_{\boldsymbol{B}1},\dots, {x}_{Bn}]$, that measure the expression of a gene for $m$ and $n$ replicates in the groups $A$ and $B$, respectively, we determine the number of values in the group $A$ that are within the range of the values of the group $B$: ${m}^{\prime }=\#\{i|\min ({\boldsymbol{x}}_{\boldsymbol{B}})\le{x}_{Ai}\le \max ({\boldsymbol{x}}_{\boldsymbol{B}})\}$, where $\#$ denotes the cardinality of a set. Similarly, we determine the number of values of the group $B$ that are within the range of the values of the group $A$: $\mathrm{and}\ {n}^{\prime }=\#\{i|\min ({\boldsymbol{x}}_{\boldsymbol{A}})\le{x}_{Bi}\le \max ({\boldsymbol{x}}_{\boldsymbol{A}})\}$. The average overlap $O$ is defined as

$$ O=\frac{1}{2}\left(\frac{m^{\prime }}{m}+\frac{n^{\prime }}{n}\right). $$

A high average overlap close to one indicates that the data points of the two groups are mixed and the gene is not differentially expressed. In contrast, a low value close to zero indicates that the groups are differentially distributed and the gene is differentially expressed. We use the average overlap as one of the measures besides average expression and fold change to investigate the properties of the genes from the reference-based simulation that were classified differently by the pseudobulk methods and the mixed models.

### Mock comparison using real data to estimate the proportion of false positives

To estimate the proportion of false positives, we performed a mock analysis using a real scRNA-seq dataset that includes PBMCs from healthy subjects, patients with flu or COVID-19 [26]. We took the 14 healthy control samples and used the metadata stored in the publicly available Seurat object (GEO accession GSE161918) to extract B cells that were labeled as singlets and had a maximum of 10% mitochondrial reads. We randomly assigned one of the two mock groups for each sample and performed statistical testing between the mock groups using each of the 18 methods to determine the DS genes. A gene was considered significant if FDR $\le$ 0.05. Before adjusting the *P*-values for multiple comparisons, we excluded the genes that were not included by all the methods (Table 1). We performed the random mock group assignment 30 times using different random seeds.

### Reproducibility

Biological measurements need to be reproducible between experiments that study the same problem but with different replicates [28]. To study the reproducibility of DS detection methods, we randomly downsampled the Liu data set [26] from the reference-based simulation that consists of 10 replicates per condition and 20 000 cells in total. In line with the rest of the reference-based simulations, the minimum number of replicates per condition was four. In addition, we also considered the imbalanced version of the same data set for which we downsampled cells from the replicates, with the remaining proportion of cells ranging with even intervals from 20 to 100%. We repeated the downsampling of the replicates 50 times for both the balanced and imbalanced data sets, generating in total 100 data sets. To measure reproducibility, we calculated Spearman’s rank correlation between each pair of the 100 data sets using the nominal *P*-values as input.

## Results

### Simulation based on a reference-free negative binomial generative model

We simulated data based on a reference-free negative binomial generative model from the original paper of the NEBULA method (see the section titled, ‘Simulation using a reference-free negative binomial generative model’). To benchmark the methods in a way that is not limited to a single *P*-value cutoff, we calculated the AUROC for each method and cluster (see the section titled, ‘Performance evaluation’). The AUROC values were, on average, highest for the pseudobulk methods, followed by the naïve and latent variable methods. The number of cells and samples did not have a noticeable impact on the superiority of the method types (Figure S1, see Supplementary Data available online at https://academic.oup.com/bib).

**Figure 1:**
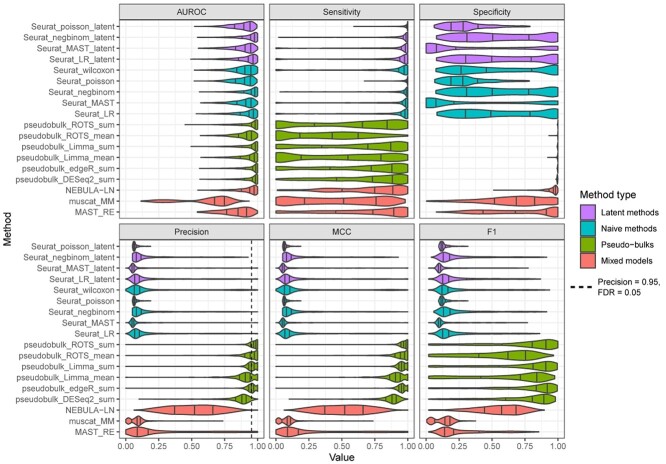
Results of the simulation based on a reference-free negative binomial generative model. Each boxplot shows values for 1280 simulated datasets with varying data properties.

In addition to the AUROC, we calculated the sensitivity, specificity, precision, F1 score and MCC using FDR of 0.05 as a cutoff to define the positives and negatives. Overall, the sensitivity was higher for the naïve methods and the latent methods compared to the pseudobulk methods and the mixed models, and it increased when the number of samples increased with all the methods, as expected (Figure S2, see Supplementary Data available online at https://academic.oup.com/bib). However, the pseudobulk methods generally provided significantly better precision and specificity compared to all other method types (Figures S3 and S4, see Supplementary Data available online at https://academic.oup.com/bib). When considering precision (Figure S4, see Supplementary Data available online at https://academic.oup.com/bib) as the inverse of FDR (FDR = 1 – Precision), the pseudobulks were the only methods that were able to achieve FDR values that were close to the expected FDR of 0.05. The F1 score (Figure S5, see Supplementary Data available online at https://academic.oup.com/bib) is the harmonic mean of sensitivity and precision that amplifies the impact of small values. Although the naïve methods achieved excellent sensitivity, their weak precision caused the F1 scores to be small, making the pseudobulks clearly the best methods based on the F1 score. The MCC is preferable to F1 score and accuracy for assessing overall performance when the binary labels (DS and non-DS) are imbalanced [29], which is often the case in gene expression data. The MCC values (Figure S6, see Supplementary Data available online at https://academic.oup.com/bib) suggested that the pseudobulks once again outperformed the other method types. With Limma and ROTS, we also tested the effect of the aggregation method on the results, suggesting the systematically better performance of the sum over the mean aggregation (Figure 1, Figures S1–S6, see Supplementary Data available online at https://academic.oup.com/bib).

Finally, we investigated how the imbalance in the number of cells between the samples affected the performance (Figure S7, see Supplementary Data available online at https://academic.oup.com/bib). In this simulation, the imbalance had a negligible effect on the performance of all methods.

### Simulation based on a reference-based negative binomial generative model

We used the muscat R package to simulate scRNA-seq data using data from four different studies (Kang, Kallionpää, Thurman and Liu; see the section titled, ‘Simulation using a reference-based negative binomial generative model’), to study the effects of different DS types, including changes in the mean expression, changes in the proportions of low and high expression-state components (DP), changes in modality and changes in both proportions and modality (DB). In total, 54 cell populations (clusters) were used in the benchmarking.

**Figure 2:**
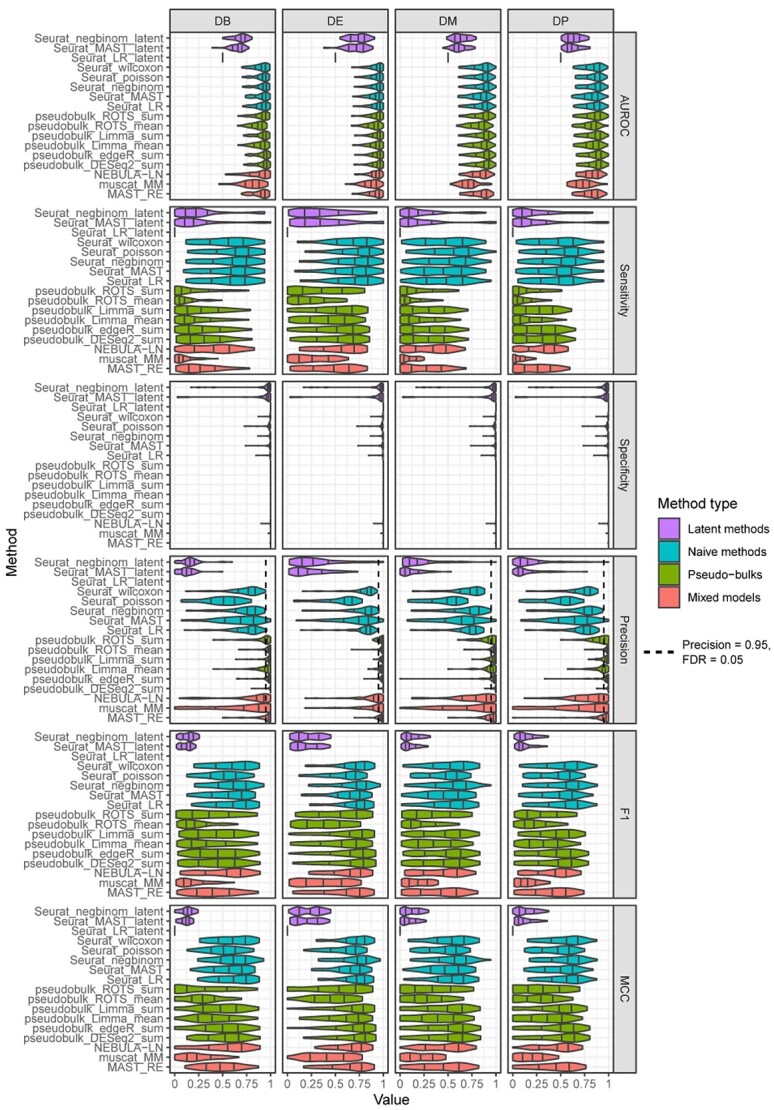
Results of the simulation based on a reference-based negative binomial generative model. Each box plot includes performance values for 54 cell populations (clusters). The rows signify the four different differential states: changes in both proportions and modality (DB), changes in the mean expression (DE), differential modality (DM) and changes in the proportions of low and high expression-state components (DP). The columns group the results by the four different performance metrics: Area under the receiver operating characteristic (AUROC) curve, sensitivity, specificity and precision. Seurat_poisson_latent was left out from the results due to its high failure rate for the simulation.

We first calculated the AUROC for each method and cluster and grouped the results by the DS type (Figure 2). These results indicate that the DS type did not have a notable impact on the ranking of the methods. Unsurprisingly, the performance scores for the DE type were consistently higher than for the three other DS types, which contained more subtle transcriptomic differences between the groups than the DE genes. The pseudobulk methods and the naïve methods achieved higher performance than the latent models and the mixed models. The latent models were clearly the weakest-performing models. The mixed models had considerable variation between their performances: MAST_RE achieved slightly better overall performance compared to NEBULA-LN, whereas muscat_MM was inferior with all four data types. Again, the pseudobulk aggregation by summing performed generally better than the mean aggregation.

In addition to the AUROC, we calculated the sensitivity, specificity, precision, F1 score and MCC using FDR of 0.05 as a cutoff to define the positives and negatives (Figure 2). The results suggested that the naïve methods provided the best sensitivity from the method types, followed by the latent variable models. However, their specificity and precision were worse compared to the pseudobulk methods and the mixed models (Figure 2). In other words, the pseudobulk methods and the mixed models were able to effectively minimize the number of false positives, whereas a significant proportion of the findings, generally 25% (precision 0.75), found by the naïve methods and the latent variable models were false. The estimated FDR levels of the pseudobulks were in concordance with the expected FDR of 0.05, but the other method types achieved inflated FDR levels. Overall, the precision and specificity were higher for the pseudobulk methods than for the mixed models. The F1 score and MCC suggested that the pseudobulks, mixed models and naïve methods performed similarly. The F1 score intensified the low sensitivity values of the pseudobulk methods, explaining the discrepancy in the results in the reference-free simulation (Figure 1). The results remained similar when investigating the impact of the number of cells and samples on the performance (Figures S8–S13, see Supplementary Data available online at https://academic.oup.com/bib).

**Figure 3:**
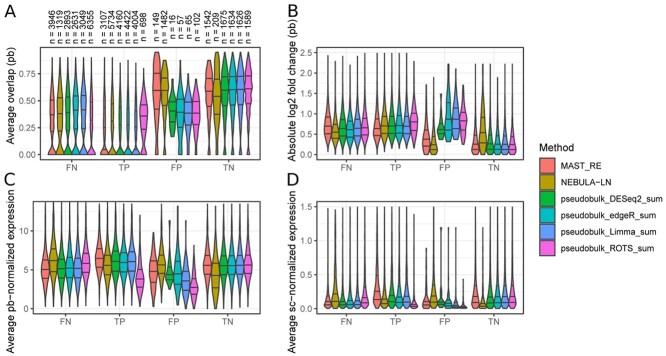
Analysis of the genes that were differently detected between the pseudobulk methods and two top-performing mixed models in the negative binomial simulation. (**A**) Average overlap was calculated by counting in each group how many pseudobulk (pb) normalized data points (samples) were within the range of the values of the other group, divided by the number of samples in the group and then taking their average. (**B**) Absolute value of the log2-transformed fold change calculated between the pseudobulk normalized gene expression values of the two groups. We added a pseudocount value of 1 to each mean expression when calculating their fold change. (**C**) Average pseudobulk normalized gene expression. The pseudobulk normalization was performed using the normalization that was used for Limma and ROTS (Table 1). (**D**) Average single-cell (sc) normalized gene expression. The single-cell normalization was performed using the normalization method that muscat simulator uses. To make the boxplots more readable, we removed the outliers for (**B–D**).

To take a closer look at the two best-performing mixed models (NEBULA-LN and MAST_RE) and the four pseudobulk methods that use the sum aggregation, we studied the genes that behaved differently among the six methods (Figure 3). We defined a metric called the average overlap to measure the differential distribution of data points between two groups (see the section titled, ‘Average overlap to measure differential distribution of points between conditions’) for which a low value indicates DE and a high value no changes in expression. When investigating the average overlaps of the gene-wise distributions between the sample groups, the false positives of the pseudobulk methods had a small overlap (from 0% to 35%) compared to true negatives (on average above 50%), suggesting that the false positives of the pseudobulk methods occurred due to errors in normalization or random chance in the simulation (Figure 3A). The false positives of the mixed models did not show such a trend, and their average overlap values were relatively high (above 0.5) and similar to those of the true negatives (Figure 3A), suggesting that the false positives of the mixed models occurred due to faults in the DS modeling by the methods themselves. The fold changes indicated that the false positives of the pseudobulk methods also had a higher fold change than the mixed models or the true negatives and they were comparable to the true positives (Figure 3B). However, the false positives of the pseudobulk methods had generally lower average expression than the mixed models or the true positives (Figure 3C and D).

Finally, we investigated how the imbalance of the number of cells affected the performance (Figure S14, see Supplementary Data available online at https://academic.oup.com/bib). In general, the AUROC values of the methods were noticeably lower in the imbalanced datasets, but especially the sensitivity of the methods decreased in the imbalanced datasets. The imbalance did not affect the superiority of the method types.

### Mock comparison with real data

**Figure 4:**
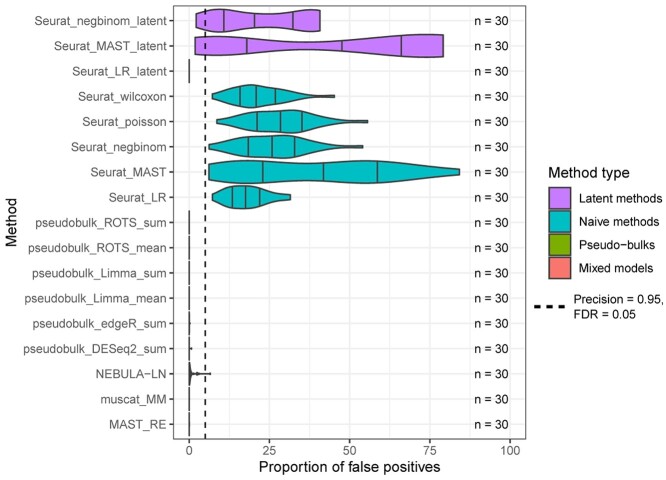
Mock analysis using real data to estimate the proportion of false positives. The mock analysis was performed by segregating good-quality B cells from a COVID-19 dataset [26] that consists of 14 healthy control subjects and by randomly assigning one of the two mock groups for each subject. The assumption is that no genes with differential states should be found between the random mock groups. The random sampling was repeated 30 times. Seurat_poisson_latent was left out from the results due to its high failure rate (29/30) for the mock data. The dashed vertical line at 5% denotes the expected maximum proportion of false positives with an FDR threshold of 0.05.

To further estimate the proportion of false positives for each method in a real experimental setting, we carried out a mock comparison by randomly dividing 14 healthy subjects from a COVID-19 study into 2 mock groups. The results suggest that the naïve methods that did not account for subjects in any way and the latent methods were subject to a high number of false positives (Figure 4). In contrast, the pseudobulk methods and the mixed models generally produced small numbers of false positives, which are in concordance with the expected FDR of 0.05. This is in accordance with the simulation results in the section titled, ‘Simulation based on a reference-free negative binomial generative model’. The logistic regression model of Seurat, which models the subjects as a latent variable, did not find any false positives, but this is likely due to the method’s inability to produce any positive findings for some data types, such as the reference-based negative binomial simulation (see the section titled, ‘Simulation based on a reference-based negative binomial generative model’).

### Reproducibility analysis with simulated data

**Figure 5:**
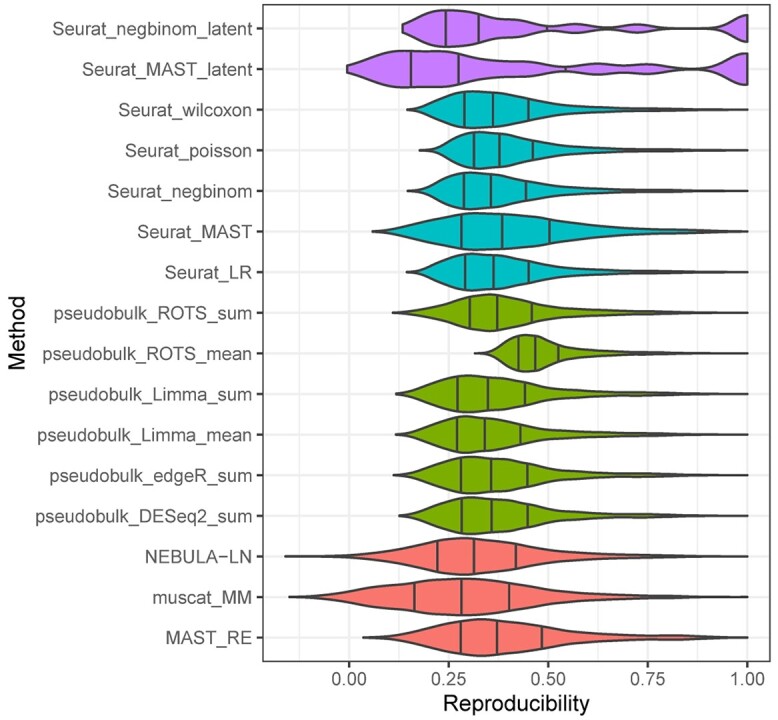
Analysis to assess reproducibility of the DS analysis methods. Spearman’s correlation between *P*-value lists. Spearman’s correlation could not be calculated for Seurat_LR_latent because all its *P*-values were constant. Seurat_poisson_latent crashed during the DS analysis runs. Each violin plot includes correlation values between all pairs (with symmetrical pairs excluded) of the 100 subsets of the Liu data. The subsets were generated by sampling replicates without replacement from the data set that consists of 10 replicates per condition and 20 000 cells.

To investigate the reproducibility of the 18 DS detection methods, we subsampled replicates from the Liu data set 100 times (see the section titled, ‘Reproducibility’) and analyzed the correlation of the *P*-value lists using Spearman’s rank correlation coefficient. The results (Figure 5) suggested that the latent methods produced the least reproducible predictions. Overall, the reproducibility of the pseudobulk methods, naïve methods and mixed models was similar. However, the pseudobulk method that uses ROTS in statistical testing and the mean aggregation achieved significantly higher reproducibility compared to the rest of the pseudobulk methods. This likely occurs due to the built-in reproducibility optimization of ROTS, which is not used by any of the other methods. In addition, MAST_RE achieved markedly higher reproducibility than muscat_MM, NEBULA-LN and many pseudobulk methods.

## Discussion

Finding DS between conditions from scRNA-seq data involves performing statistical testing between two or more groups of cells for each cell type separately. scRNA-seq experiments increasingly include multiple subjects or biological replicates to confirm that the transcriptomic changes are prevalent in groups and not only in single subjects. This requires specialized tools due to the hierarchical structure of the data. Cells from the same subject often have more similar gene expression profiles, which violates the statistical independence assumption of the basic statistical tests.

This issue has been already addressed in recently published studies that have proposed new, improved methods for the DS analysis of multisubject scRNA-seq data [6, 8, 9, 16]. The two approaches that currently seem the most promising are the pseudobulk methods that aggregate counts from each cluster and sample and the mixed models that model the subjects as a random effect. Both have been demonstrated to decrease the number of false positives compared to regular statistical tests, such as the Wilcoxon rank sum test, that naïvely assumes the cells are statistically independent [8, 10]. However, no attempts have yet been made to compare these tools in the same work.

In this article, we compared 18 tools for the DS analysis of multisubject scRNA-seq data. These methods included both pseudobulk methods and mixed models, but also naïve single-cell methods that do not model the subjects in any way, as well as methods that model the subjects as a latent variable. Our benchmarking framework included both simulated and real data. For the simulation, we considered both a reference-free negative binomial generative model and a reference-based negative binomial generative model. The reference-free generative model simulated genes with changes in the mean expression (DE). The reference-based negative binomial generative model simulation was performed using the muscat R package, which is currently the only simulator that can simulate multisubject scRNA-seq data with four different DS types. Finally, we performed a mock comparison using 14 healthy control subjects from a large COVID-19 dataset, [26] which enabled us to estimate the proportion of false positives for each method.

The results indicated that the naïve methods were indeed subject to a higher rate of false positives than the pseudobulk methods and the mixed models. This conclusion is supported by all our analyses. While the naïve models generally provided higher sensitivity, this benefit was negated by the lower precision and specificity. In other words, the naïve models reported a lot of findings, but a large proportion of these were false positives. Although the AUROC results suggested that the *P*-values of the naïve methods accurately ranked the positives before the negatives, the main issue was that the *P*-values were poorly calibrated. With an FDR of 0.05 as a cutoff to define the positives and negatives, the methods can be expected to find at most 5% false positives from all positives. The naïve methods found high proportions of false positives, up to 40% in the mock comparison and the simulations. In the simulation, the precision values of the pseudobulk methods were in better accordance with the FDR cutoff than the precision values of the mixed models.

We observed notable variation in the performances of the mixed models. Of the three mixed models that we considered in our comparison, MAST_RE and NEBULA-LN achieved considerably better overall performance than muscat_MM. However, the performances of the pseudobulk methods were mostly similar. Our results suggested that the pseudobulk aggregation by calculating the mean of single-cell normalized data provided inferior performance compared to the sum approach that cumulatively sums the count values and then uses bulk RNA-seq normalization. This is in line with at least one previous study that found that the sum aggregation outperformed the mean aggregation [6].

We investigated how the number of cells and samples affected the performance of the methods. An earlier study [8] found that the pseudobulks performed worse than the mixed models when the number of samples was small. The same paper also suggested that the pseudobulks would perform worse than the best-mixed model (MAST_RE) when the samples have an uneven distribution for the number of cells. However, we were unable to validate these findings.

The popular Seurat pipeline includes four statistical tests that allow for the incorporation of several latent variables in the models. The latent models test if the observed DE change between the conditions can be explained by the difference in one or several variables. According to the package manual, this is recommended if the data contains batch effects in the DS analysis, but no instructions are provided for any other variables. As of Seurat v4.1, using latent variables is currently the only way to account for the subjects in the DS analysis with Seurat. Our results strongly suggest against their use in the DS analysis when the subject is included as a latent variable. The latent models performed generally even worse than their naïve counterparts. A recent study came to the same conclusion when they used ComBat to correct the data for the subject effect before the DS analysis [8, 30]. However, the latent models might still be appropriate when modeling batch effects or other variables as latent variables.

Our comparison includes limitations and details that can cause bias in the results. First, the reference-free simulation was originally presented in the original paper of NEBULA-LN, which is one of the methods compared in this article. The simulation uses the same generative model that is used by the NEBULA mixed model. Therefore, NEBULA could have an unfair advantage over the other mixed models in the comparison. Secondly, our comparison did not investigate the role of prepreprocessing, such as normalization or clustering. In this article, we focused solely on benchmarking DS detection methods for multisubject, multicondition data. More versatile comparisons are still needed to investigate the complex relationship between the different analysis steps of which the DS detection is only one step [31]. While our simulation framework included data sets with an imbalanced distribution of cells between the subjects, we did not consider the most extreme scenarios where the same types can be extremely rare in some subjects but abundant in other subjects.

Our findings opened many interesting research questions that require further investigation in future studies. Our results suggested that the mean aggregation generally outperformed the sum aggregation. Investigating why exactly the sum aggregation performed better was not investigated in this study. This would require adjusting the normalization workflows of the pseudobulk methods to test, for example, whether the TMM normalization [32] is a beneficial step. Interestingly, however, the mean aggregation provided better reproducibility and precision in the reference-free simulation for ROTS. The second interesting observation was the significant variation in the performance of the three mixed models. Our results suggested that muscat_MM was inferior to NEBULA and MAST_RE. The inferiority of muscat_MM was mainly attributable to its low sensitivity. In the original study on muscat, the results suggested in the same way that muscat_MM achieved lower sensitivity than the pseudobulk methods. It would be useful to investigate in future studies what exactly causes the low sensitivity of muscat_MM. This would require testing different configurations of the mixed modeling and data transformation. However, muscat_MM uses linear mixed modeling (lme4) and voom transformation, which differs from the other two mixed models.

To conclude, we performed a comprehensive comparison to benchmark 18 methods for DS analysis of multisubject scRNA-seq data. Our results suggest that the pseudobulk methods and the mixed models that model subjects as a random effect were superior compared to the naïve single-cell methods that do not model the subjects in any way. We also recommend not to perform DS analysis using Seurat’s statistical tests so that the subjects are modeled as a latent variable. Overall, the pseudobulk methods outperformed the mixed models. If the user wants to achieve high specificity and precision at the risk of losing some true positives, we recommend the pseudobulk ROTS with the sum aggregation. If sensitivity is more important than the false-positive results, then we recommend the pseudobulk methods Limma, DESeq2 or edgeR combined with the sum aggregation. We recommend that scRNA-seq analysis pipeline developers should begin to include pseudobulk methods and mixed models in their pipelines. To facilitate DS analysis of multisubject scRNA-seq data, the codes that implement all the methods in this article are freely available online (https://github.com/elolab/multisubjectDSanalysis).

## Author’s contributions

L.L.E. and S.J. conceived the study. L.L.E., S.J. and J.S. designed the study. J.S. and S.J. implemented the study. J.S. wrote the manuscript. L.L.E. and S.J. commented the manuscript. L.L.E. supervised the study. S.J. and J.S. share the first authorship.

**Key Points**

- Naïve single-cell differential states analysis methods are subject to high proportions of false positives when analyzing data with multiple biological replicates.
- Latent variable models are not effective in reducing false discoveries when accounting for biological replicates.
- Pseudobulk methods and mixed models that account for biological replicates as a random effect are effective in reducing false discoveries.
- Overall, our results suggest that pseudobulk methods outperform mixed models.

## Supplementary Material

BiB-Supplementary_File-Benchmarking_27_04_2022_JS_bbac286

## Data Availability

Codes for the 18 DS analysis methods along with the benchmarking codes are available at https://github.com/elolab/multisubjectDSanalysis.

## Notes

**Sini Junttila** is a postdoc at the Medical Bioinformatics Center at Turku Bioscience Centre. Her expertise is in analysis of scRNA-seq and epigenomics data.

**Johannes Smolander** is a PhD student at the University of Turku specializing on single-cell RNA-seq data analysis. He works at the Medical Bioinformatics Center at Turku Bioscience Centre.

**Laura L. Elo** is a professor of Computational Medicine and the Head of the Medical Bioinformatics Centre, University of Turku, Finland. Her main research interests include computational biomedicine and bioinformatics.
