# Supplementary material for: Benchmarking methods for detecting differential states between conditions from multi-subject single-cell RNA-seq data
Source: Brief Bioinform. 2022 Jul 25;23(5):bbac286. doi: 10.1093/bib/bbac286 (PMC9487674; doi:10.1093/bib/bbac286)
Supplement: BiB-Supplementary_File-Benchmarking_27_04_2022_JS_bbac286 [file bib-supplementary_file-benchmarking_27_04_2022_js_bbac286.zip › BiB-Supplementary_File-Benchmarking_methods_for_detecting_differential_data_27_04_2022_JS_bbac286.pdf]

Supplementary File

Supplementary Figure 1

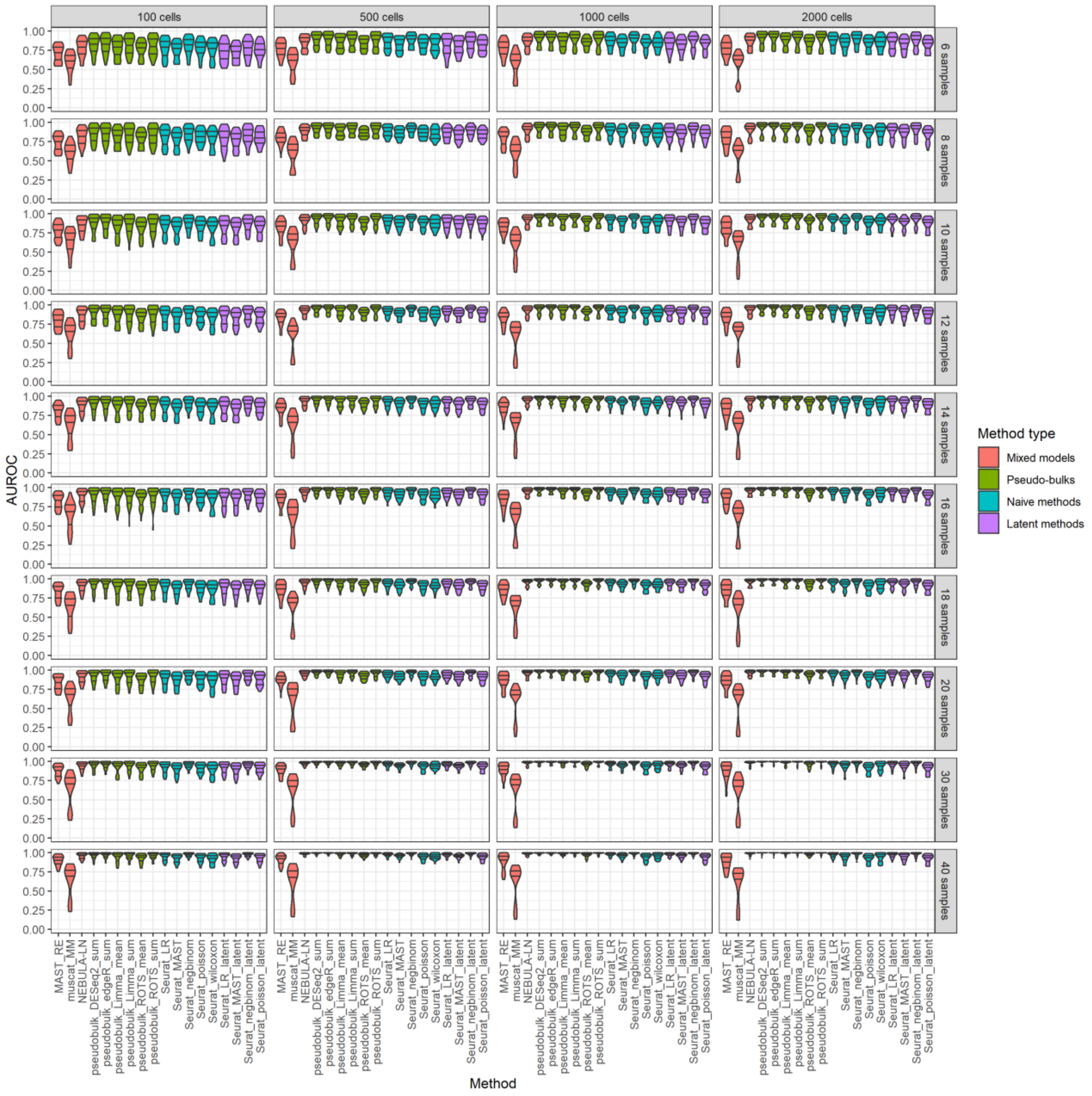

Supplementary Figure 1. Area Under Receiver Operating Characteristic (AUROC) values for the reference-free negative binomial generative simulation. Each violin plot includes results of 32 data sets simulated using four cell overdispersion values (0.1,1,10,100), four sample overdispersion values (0.05, 0.1, 0.2, 0.5) and two cell number distributions (imbalanced=negative binomial distribution, balanced=poisson distribution). The results are grouped by the average number of cells per subject (columns) and the number of subjects per dataset (e.g. 6 means 3 vs. 3). Each dataset includes 2000 genes, of which 100 are defined as positives by having $2 \geq \text{logFC} \geq 0.5$ between the two conditions, and the rest 1900 have logFC=0. The simulation is based on the simulation in the original paper of the NEBULA method. To estimate AUROC, we used the uncorrected p-values as the predicted values. We considered only those genes that were not filtered out by any of the methods.

Supplementary Figure 2

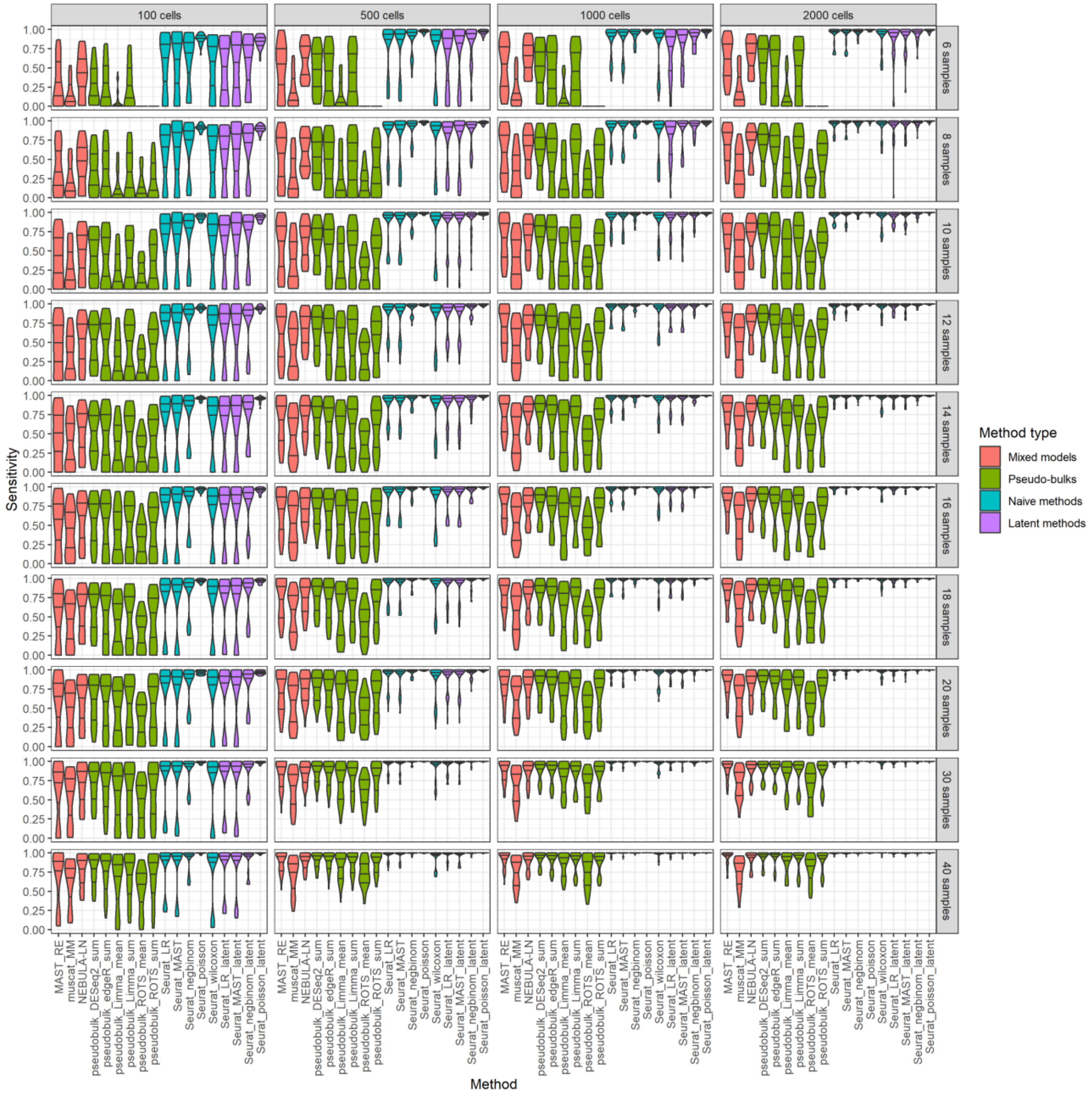

Supplementary Figure 2. Sensitivity values for the reference-free negative binomial generative simulation. Each violin plot includes results of 32 data sets simulated using four cell overdispersion values (0.1,1,10,100), four sample overdispersion values (0.05, 0.1, 0.2, 0.5) and two cell number distributions (imbalanced=negative binomial distribution, balanced=poisson distribution). The results are grouped by the average number of cells per subject (columns) and the number of subjects per dataset (e.g. 6 means 3 vs. 3). Each dataset includes 2000 genes, of which 100 are defined as positives by having 2 ≥ logFC ≥ 0.5 between the two conditions, and the rest 1900 have logFC=0. The simulation is based on the simulation in the original paper of the NEBULA method. To define the positive and negative DE genes for each method, we adjusted the p-values using the Benjamini-Hochberg method (also known as the FDR method) and used FDR=0.05 as the threshold. We considered only those genes that were not filtered out by any of the methods.

Supplementary Figure 3

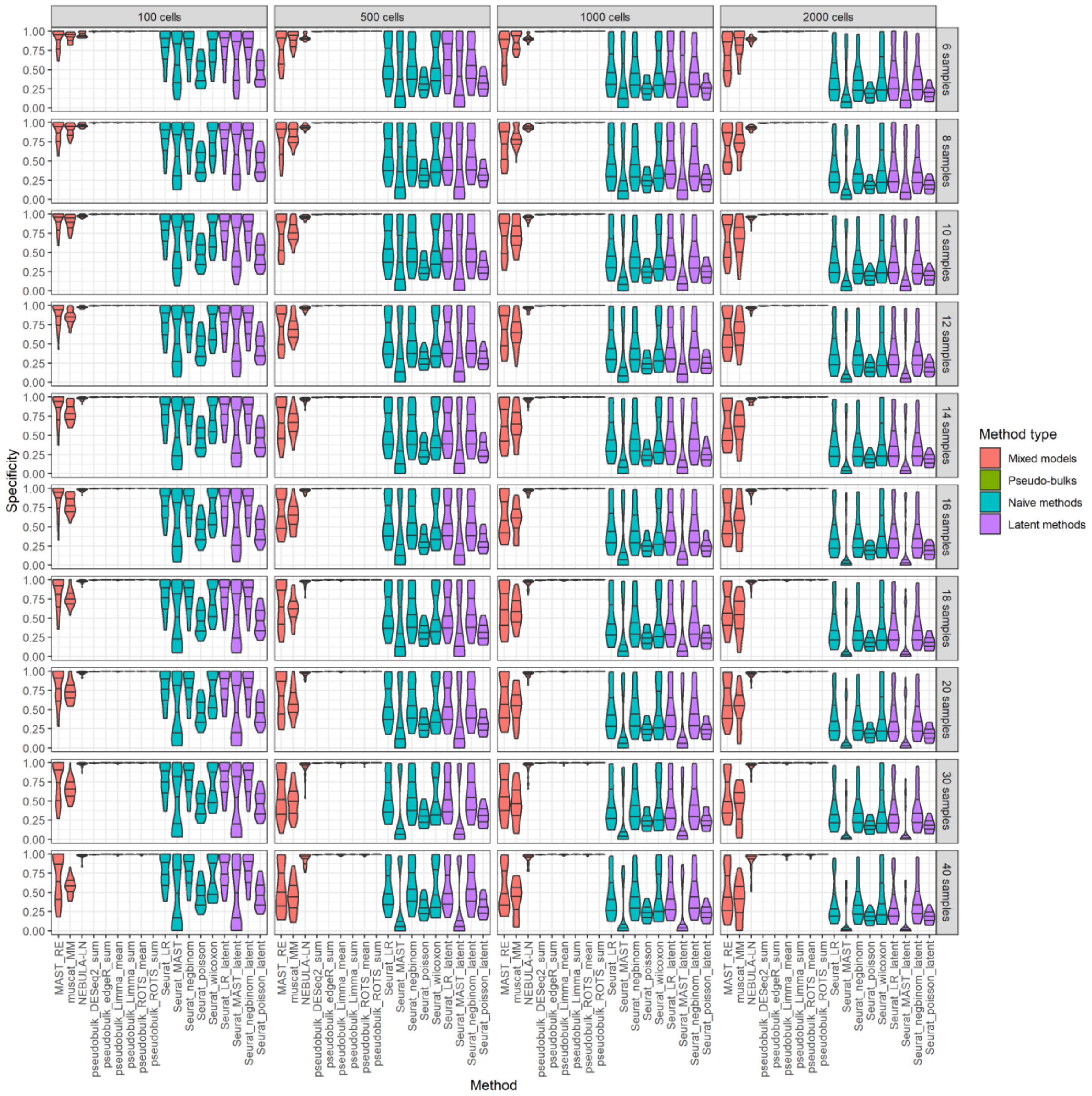

Supplementary Figure 3. Specificity values for the reference-free negative binomial generative simulation. Each boxplot includes results of 32 data sets simulated using four cell overdispersion values (0.1,1,10,100), four sample overdispersion values (0.05, 0.1, 0.2, 0.5) and two cell number distributions (imbalanced=negative binomial distribution, balanced=poisson distribution). The results are grouped by the average number of cells per subject (columns) and the number of subjects per dataset (e.g. 6 means 3 vs. 3). Each dataset includes 2000 genes, of which 100 are defined as positives by having $2 \geq \text{logFC} \geq 0.5$ between the two conditions, and the rest 1900 have logFC=0. The simulation is based on the simulation in the original paper of the NEBULA method. To define the positive and negative DE genes for each method, we adjusted the p-values using the Benjamini-Hochberg method (also known as the FDR method) and used FDR=0.05 as the threshold. We considered only those genes that were not filtered out by any of the methods.

Supplementary Figure 4

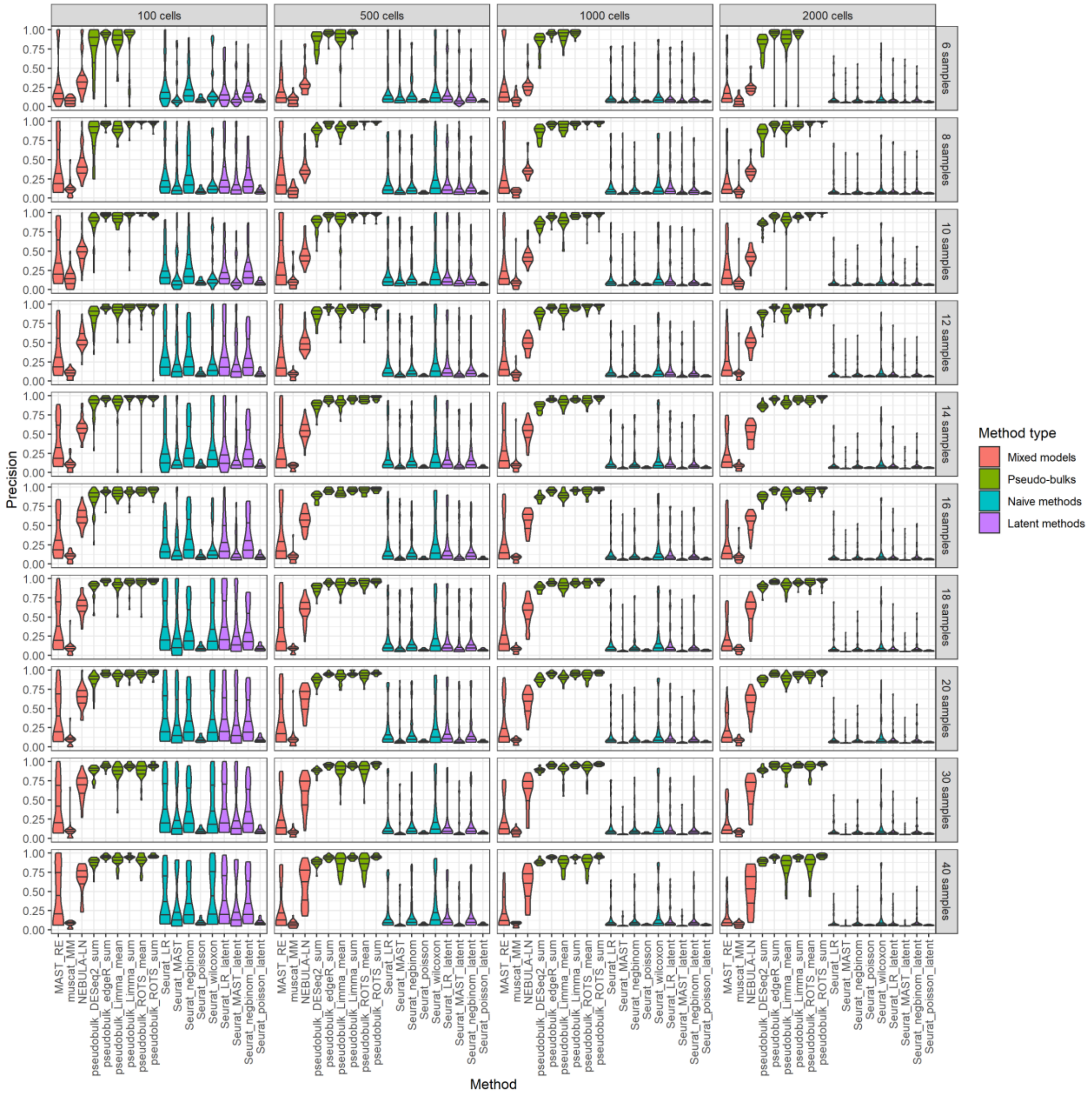

Supplementary Figure 4. Precision values for the reference-free negative binomial generative simulation. Each boxplot includes results of 32 data sets simulated using four cell overdispersion values (0.1,1,10,100), four sample overdispersion values (0.05, 0.1, 0.2, 0.5) and two cell number distributions (imbalanced=negative binomial distribution, balanced=poisson distribution). The results are grouped by the average number of cells per subject (columns) and the number of subjects per dataset (e.g. 6 means 3 vs. 3). Each dataset includes 2000 genes, of which 100 are defined as positives by having $2 \geq \text{logFC} \geq 0.5$ between the two conditions, and the rest 1900 have logFC=0. The simulation is based on the simulation in the original paper of the NEBULA method. To define the positive and negative DE genes for each method, we adjusted the p-values using the Benjamini-Hochberg method (also known as the FDR method) and used FDR=0.05 as the threshold. We considered only those genes that were not filtered out by any of the methods. If the precision could not be calculated because there were no positive genes in the results, we set the precision to zero.

Supplementary Figure 5

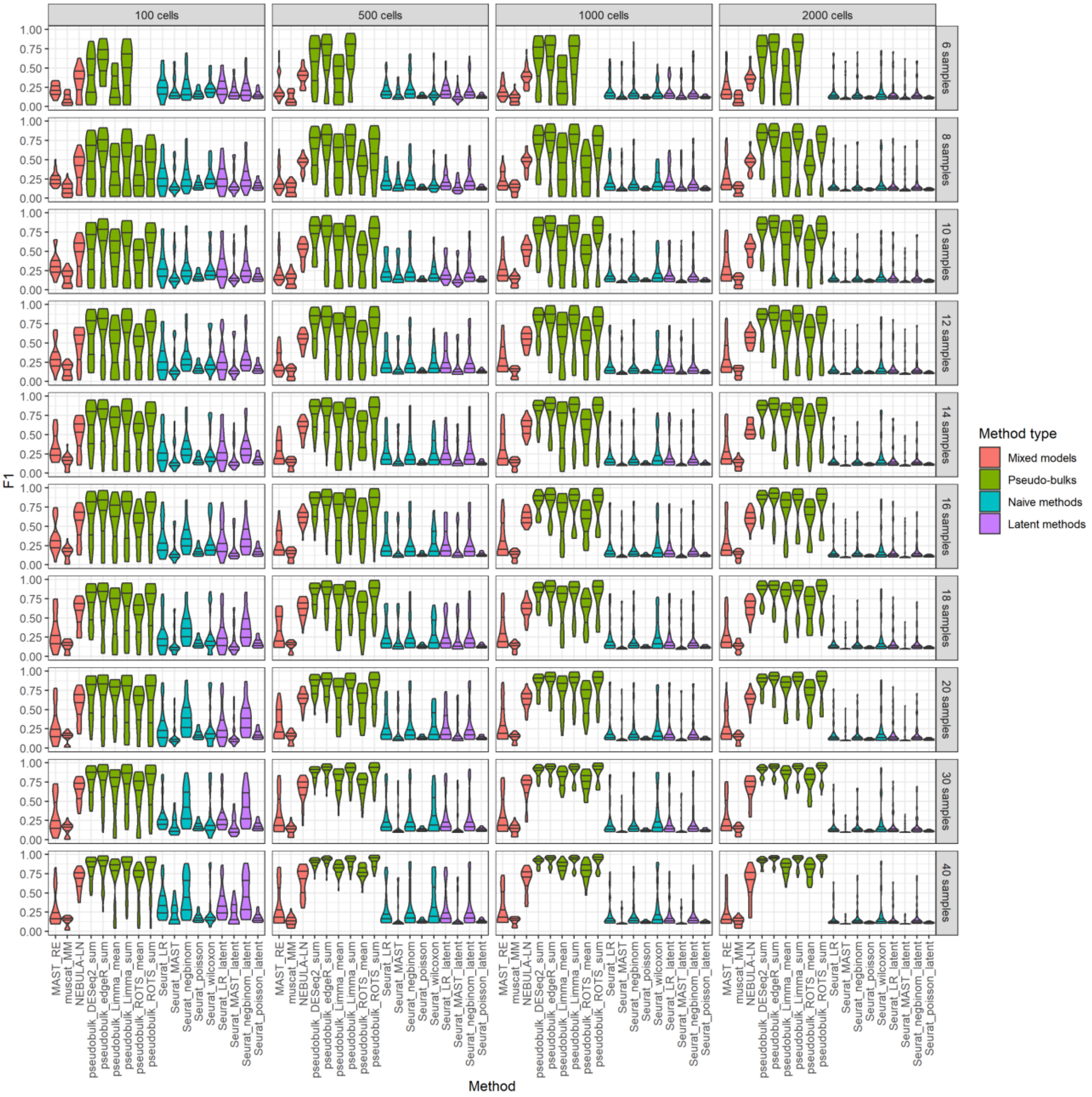

Supplementary Figure 5. F1-scores for the reference-free negative binomial generative simulation. Each violin plot includes results of 32 data sets simulated using four cell overdispersion values (0.1,1,10,100), four sample overdispersion values (0.05, 0.1, 0.2, 0.5) and two cell number distributions (imbalanced=negative binomial distribution, balanced=poisson distribution). The results are grouped by the average number of cells per subject (columns) and the number of subjects per dataset (e.g. 6 means 3 vs. 3). Each dataset includes 2000 genes, of which 100 are defined as positives by having $2 \geq \text{logFC} \geq 0.5$ between the two conditions, and the rest 1900 have logFC=0. The simulation is based on the simulation in the original paper of the NEBULA method. To define the positive and negative DE genes for each method, we adjusted the p-values using the Benjamini-Hochberg method (also known as the FDR method) and used FDR=0.05 as the threshold. We considered only those genes that were not filtered out by any of the methods. If the precision could not be calculated because there were no positive genes in the results, we set the precision to zero.

Supplementary Figure 6

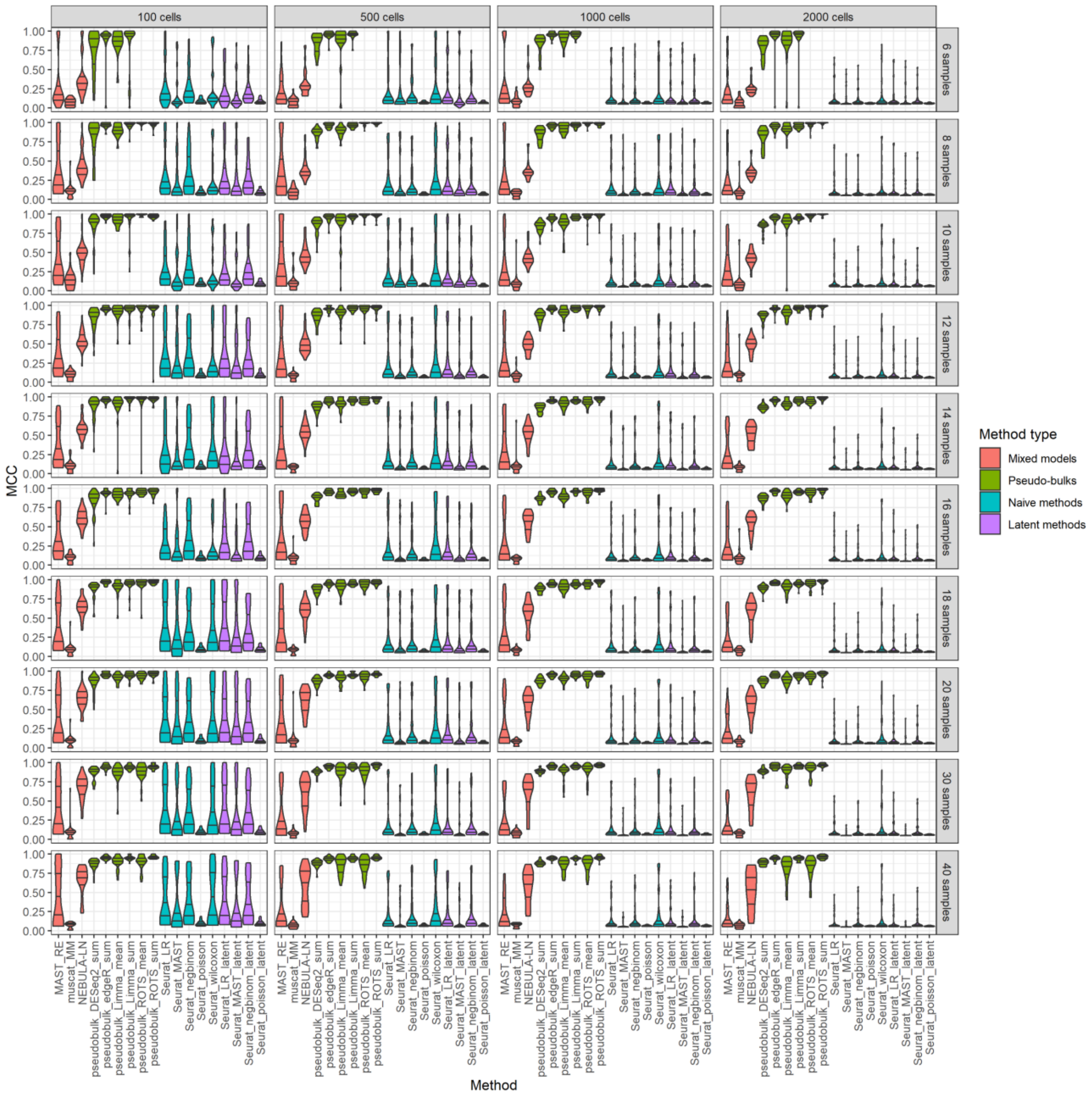

Supplementary Figure 6. Matthew's correlation coefficient (MCC) values for the reference-free negative binomial generative simulation. Each violin plot includes results of 32 data sets simulated using four cell overdispersion values (0.1,1,10,100), four sample overdispersion values (0.05, 0.1, 0.2, 0.5) and two cell number distributions (imbalanced=negative binomial distribution, balanced=poisson distribution). The results are grouped by the average number of cells per subject (columns) and the number of subjects per dataset (e.g. 6 means 3 vs. 3). Each dataset includes 2000 genes, of which 100 are defined as positives by having $2 \geq \text{logFC} \geq 0.5$ between the two conditions, and the rest 1900 have logFC=0. The simulation is based on the simulation in the original paper of the NEBULA method. To define the positive and negative DE genes for each method, we adjusted the p-values using the Benjamini-Hochberg method (also known as the FDR method) and used FDR=0.05 as the threshold. We considered only those genes that were not filtered out by any of the methods. If the precision could not be calculated because there were no positive genes in the results, we set the precision to zero.

Supplementary Figure 7

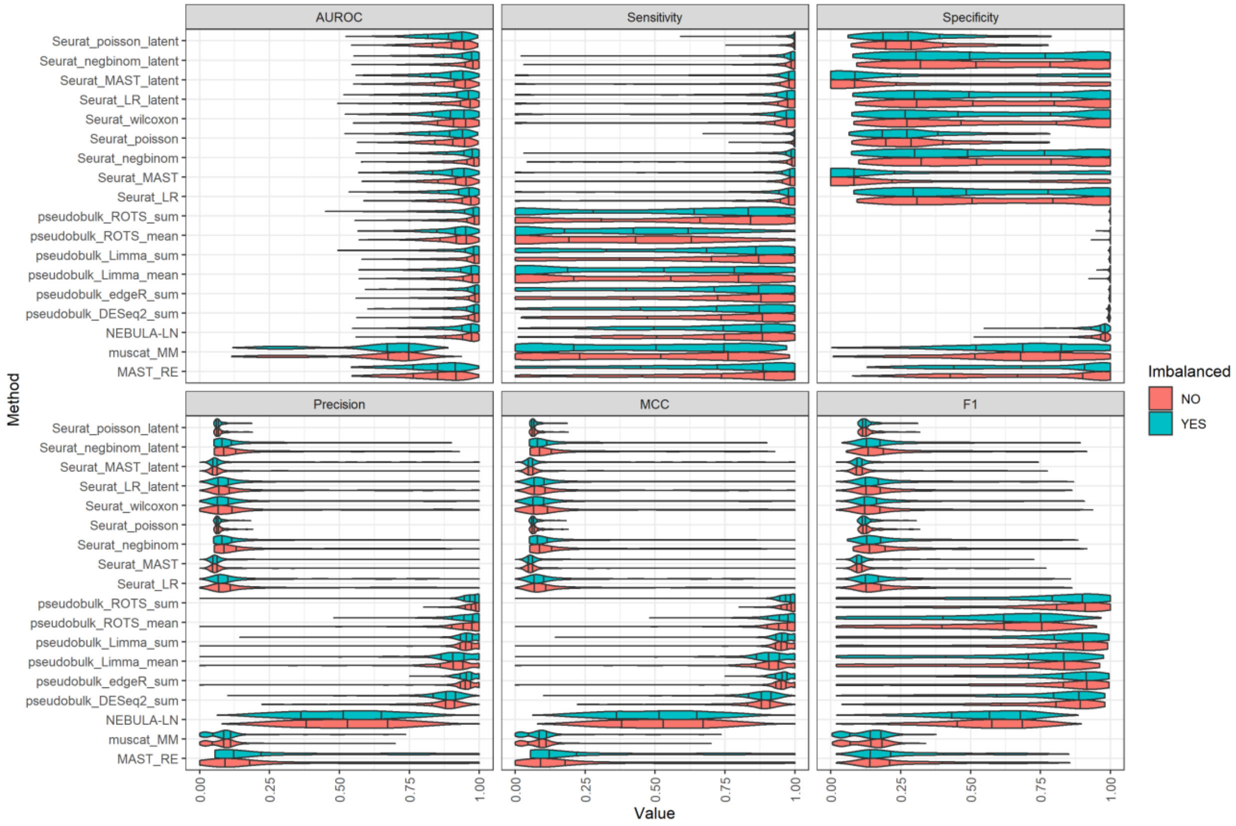

Supplementary Figure 7. Results of the reference-free negative binomial generative simulation grouped by whether the data sets were downsampled to generate an imbalance distribution of cells across the samples or not.

Supplementary Figure 8

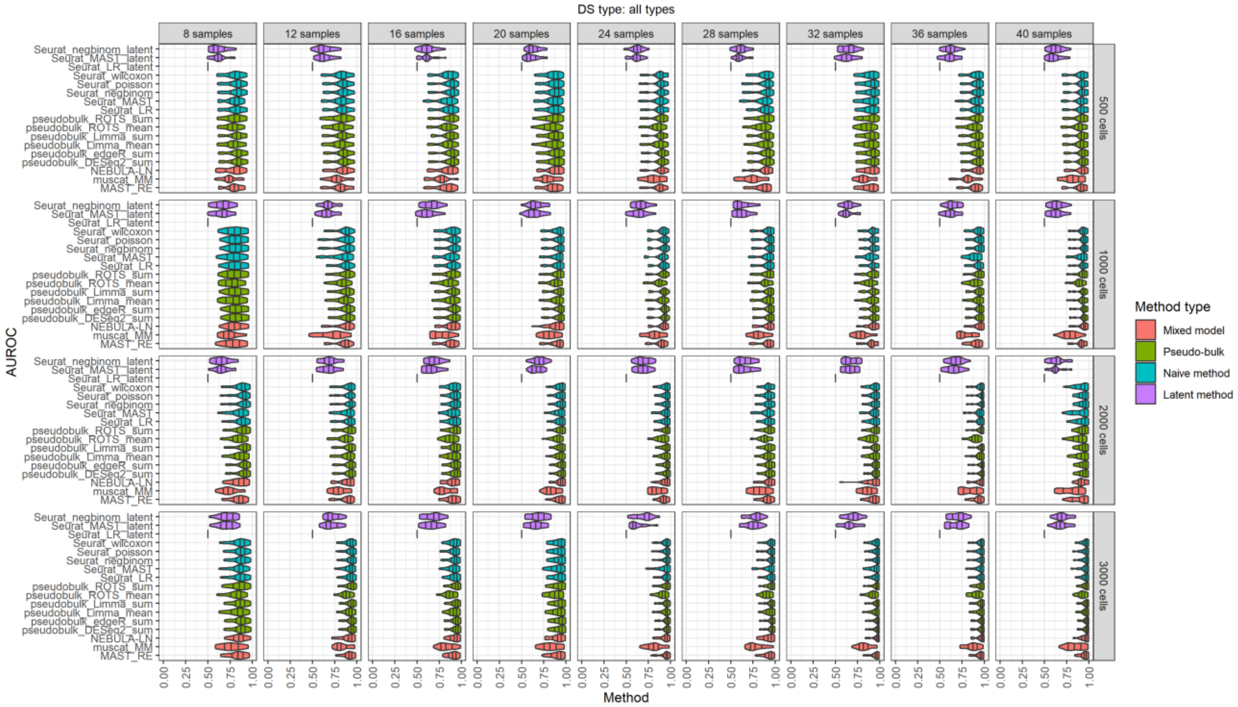

Supplementary Figure 8. Area Under Receiver Operating Characteristic (AUROC) values for the reference-based negative binomial generative simulation (muscat). The results are grouped in columns by the number of samples in the comparison and in the rows by the number of cells per subject. In this simulation, each sample includes three clusters. These results are for all four differential state (DS) types combined. See more details in **Section 2.2.1** of the manuscript.

Supplementary Figure 9

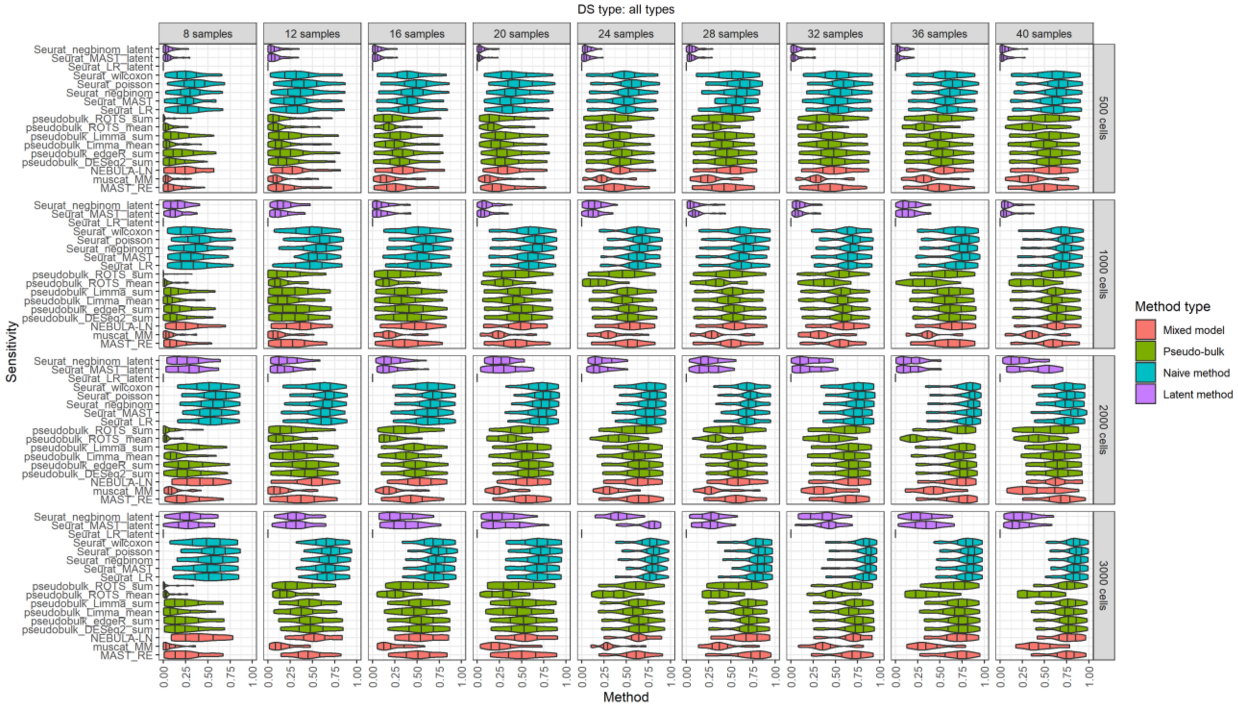

Supplementary Figure 9. Sensitivity values for the reference-based negative binomial generative simulation (muscat). The results are grouped in columns by the total number of subjects (both groups) and in the rows by the number of cells per subject. In this simulation, each sample includes three clusters. The metrics were calculated for each of the four differential state (DS) types separately, and the results are visualized here together for all four metrics. See more details in **Section 2.2.1** of the manuscript.

Supplementary Figure 10

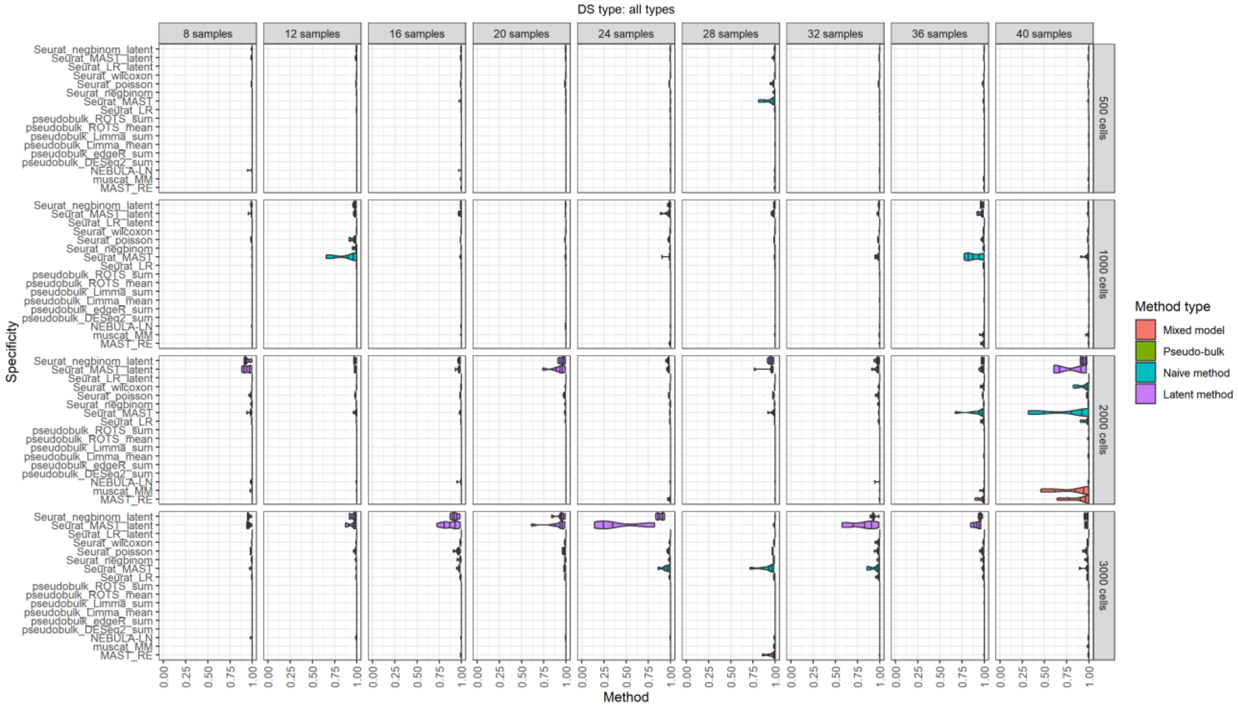

Supplementary Figure 10. Specificity values for the reference-based negative binomial generative simulation (muscat). The results are grouped in columns by the total number of subjects (both groups) and in the rows by the number of cells per subject. In this simulation, each sample includes three clusters. The metrics were calculated for each of the four differential state (DS) types separately, and the results are visualized here together for all four metrics. See more details in **Section 2.2.1** of the manuscript.

Supplementary Figure 11

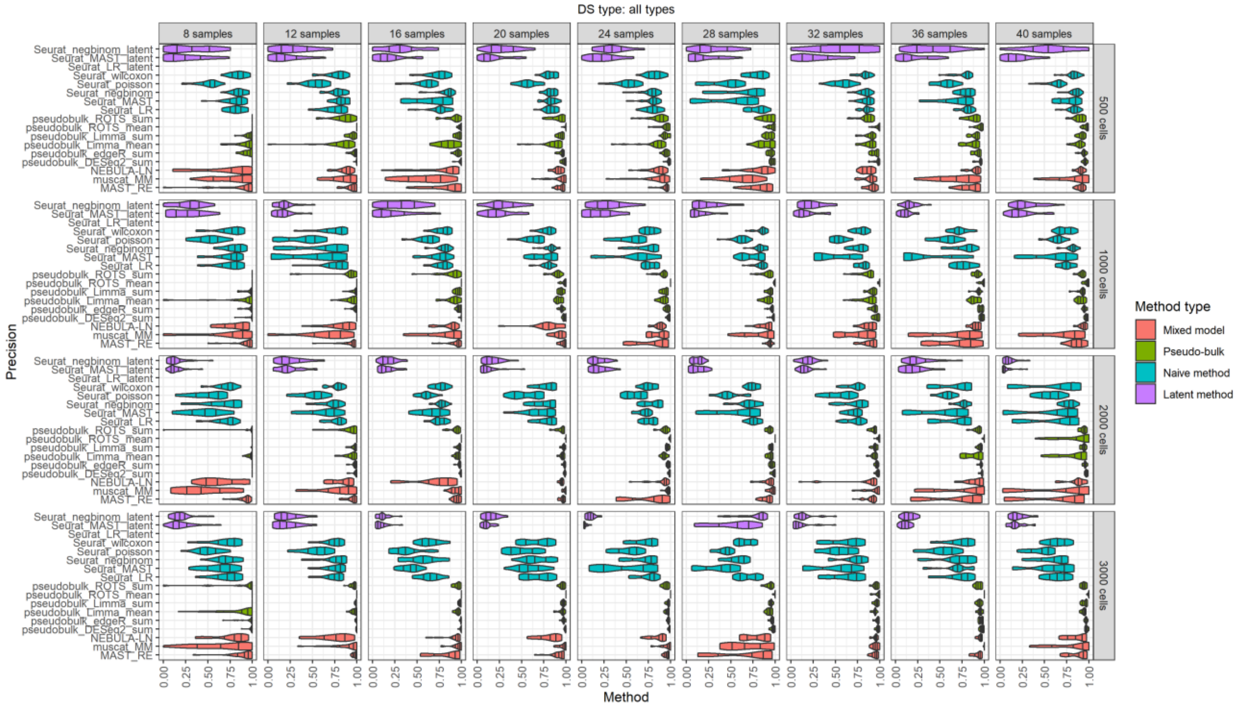

Supplementary Figure 11. Precision values for the reference-based negative binomial generative simulation (muscat). The results are grouped in columns by the total number of subjects (both groups) and in the rows by the number of cells per subject. In this simulation, each sample includes three clusters. The metrics were calculated for each of the four differential state (DS) types separately, and the results are visualized here together for all four metrics. See more details in **Section 2.2.1** of the manuscript.

Supplementary Figure 12

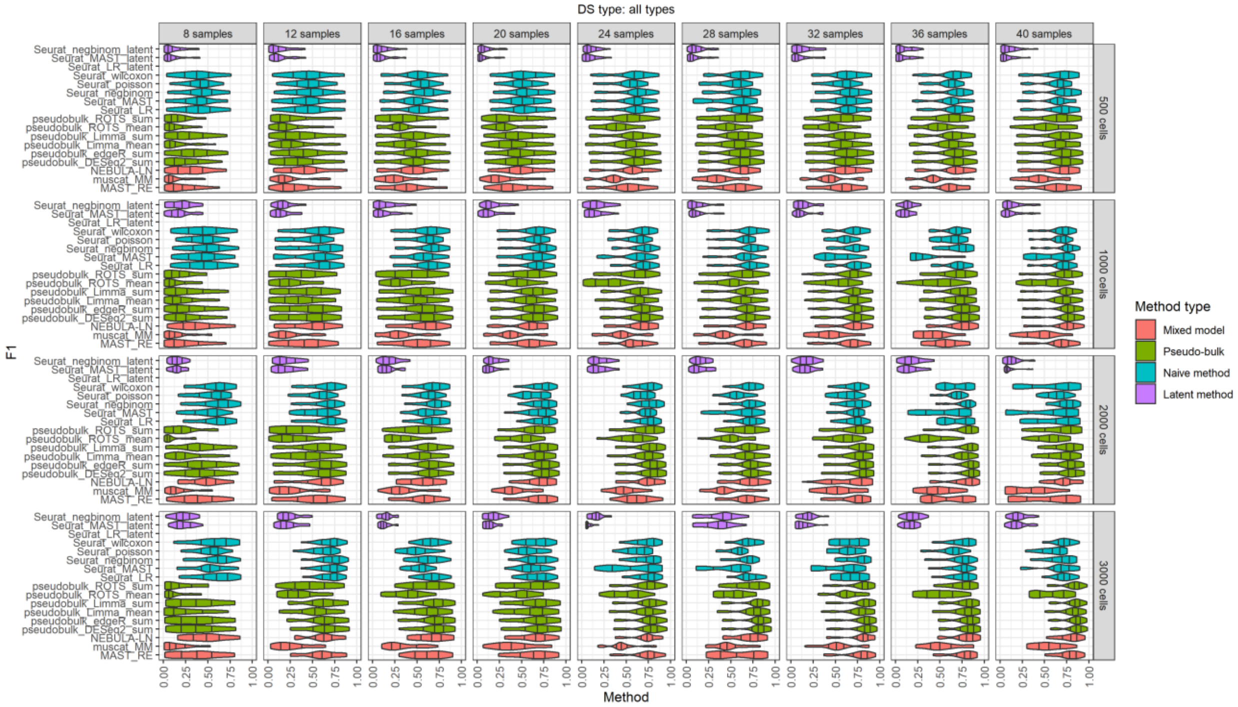

Supplementary Figure 12. F1-scores for the reference-based negative binomial generative simulation (muscat). The results are grouped in columns by the total number of subjects (both groups) and in the rows by the number of cells per subject. In this simulation, each sample includes three clusters. The metrics were calculated for each of the four differential state (DS) types separately, and the results are visualized here together for all four metrics. See more details in **Section 2.2.1** of the manuscript.

Supplementary Figure 13

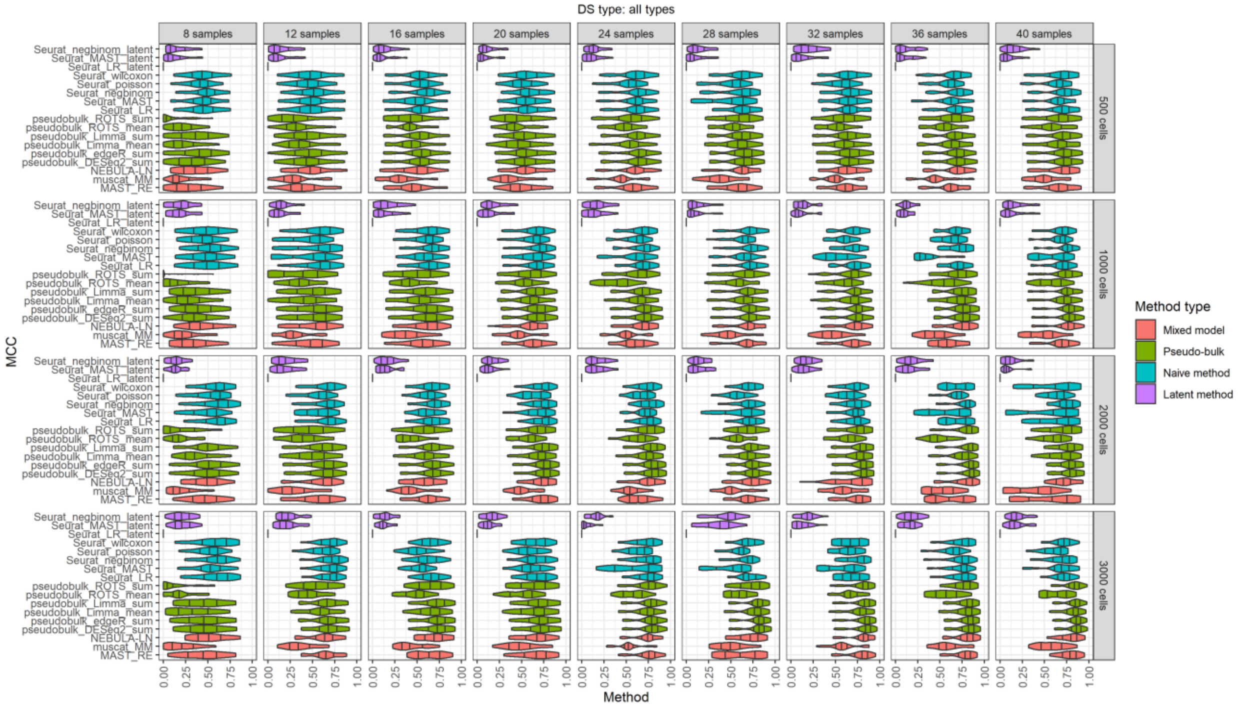

Supplementary Figure 13. Matthew's correlation coefficient (MCC) values for the reference-based negative binomial generative simulation (muscat). The results are grouped in columns by the total number of subjects (both groups) and in the rows by the number of cells per subject. In this simulation, each sample includes three clusters. The metrics were calculated for each of the four differential state (DS) types separately, and the results are visualized here together for all four metrics. See more details in **Section 2.2.1** of the manuscript.

Supplementary Figure 14

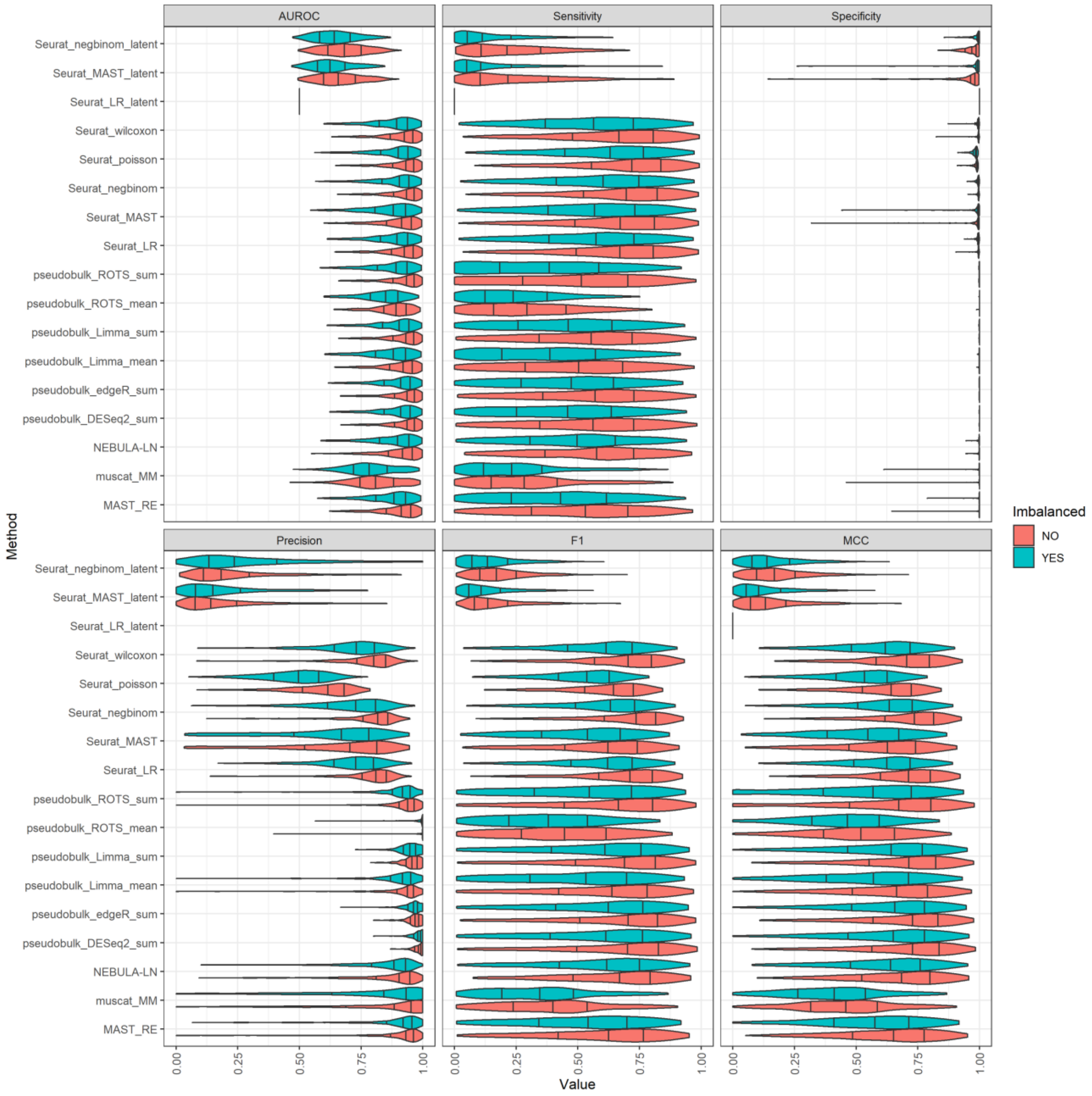

Supplementary Figure 14. Results of the cell-sample-extended reference-based negative binomial generative simulation grouped by whether the data sets were downsampled to generate an imbalance distribution of cells across the samples.
